# Effects of light therapy in the anxious-depressive clinic

**DOI:** 10.1192/j.eurpsy.2024.1107

**Published:** 2024-08-27

**Authors:** M. T. Gonzalez Salvador, R. Gutierrez-Labrador, M. I. C. Gómez-Ulla, A. I. de la Puente

**Affiliations:** ^1^Psiquiatria, Hospital Universitario Puerta de Hierro; ^2^Psiquiatria, Hospital Universitario Infanta Sofía, Madrid, Spain

## Abstract

**Introduction:**

Major depressive disorder (MDD) is defined as a mental disorder of multifactorial etiology, which presents with mood disturbance, mainly sadness associated with loss of interest or pleasure. Light therapy (LT) is a therapeutic intervention consisting of daily exposure to a light source. This study aims to evaluate the effects of LT on anxious-depressive symptomatology and sleep in a sample of patients diagnosed with depression.

**Objectives:**

This study aims to evaluate the effects of LT on anxious-depressive symptomatology and sleep in a sample of patients diagnosed with depression.

**Methods:**

Prospective case-control study, in which the cases are outpatients diagnosed with MDD and the controls are healthy individuals. Both groups underwent LT sessions and were assessed by means of validated scales, anxiety and depression symptoms before and after LT sessions, as well as changes in sleep patterns through a sleep measuring device.

**Results:**

11 cases and 18 controls were included in the study. Of the participants, 62.1% were female and 37.9% were male. The mean age of the sample was 54.03 □ 11.55 years. There were significant case differences in the pre and post LT scores of the depression scale. There were no significant differences in the changes in superficial, deep and total sleep and in the anxiety scale scores.

**Conclusions:**

In the sample analysed, LT has significant effects on the cases at the level of the depression scale.

**Disclosure of Interest:**

None Declared

